# Fostering Medical Materials Innovation

**DOI:** 10.1021/acsmaterialsau.2c00054

**Published:** 2022-10-15

**Authors:** Inge K. Herrmann, Andrea A. Schlegel

**Affiliations:** † Nanoparticle Systems Engineering Laboratory, Institute of Energy and Process Engineering (IEPE), Department of Mechanical and Process Engineering (D-MAVT), ETH Zurich, Sonneggstrasse 3, 8092 Zurich, Switzerland; ‡ Particles-Biology Interactions Laboratory, Department of Materials Meet Life, Swiss Federal Laboratories for Materials Science and Technology (Empa), Lerchenfeldstrasse 5, 9014 St. Gallen, Switzerland; § Fondazione IRCCS Ca’ Granda, Ospedale Maggiore Policlinico, Centre of Preclinical Research, Via Francesco Sforza, 35, Milan 20122, Italy; ∥ Department of Surgery and Transplantation, Swiss HPB Centre, University Hospital Zurich, Rämistrasse 100, 8091 Zurich, Switzerland

**Keywords:** interdisciplinary collaboration, advancement, technology, medicine, clinics, education

## Abstract

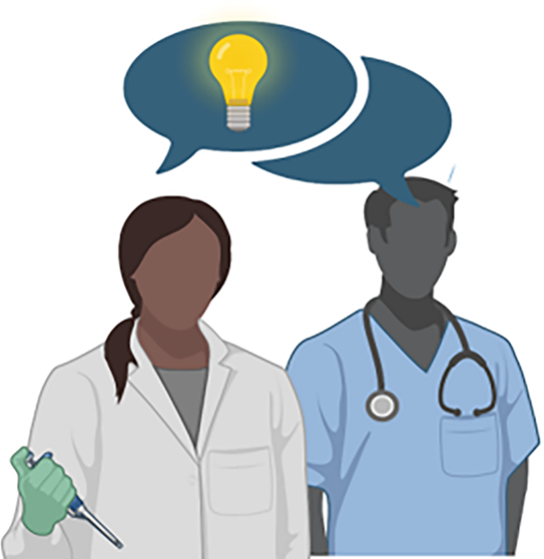

Close collaboration between basic researchers and clinicians is at the root of medical material and technology innovation. However, the distinctly different educational curricula and various boundary conditions put barriers on such interactions. This short perspective describes current challenges and provides subsequent solutions that may help research laboratories to overcome frequent hurdles and maximize interdisciplinary interactions. The involvement of various stakeholders is key to establishing an environment for barrier-free, effective collaboration, overcoming disciplinary boundaries and creating a strong source of inspiration and motivation for biomedical innovations with clinical impact.

Materials play a central role in medicine.^1,2^ The overwhelming majority of new diagnostic and therapeutic approaches are based on materials. The potential for innovation^3^ at the intersection of engineering and medicine appears almost limitless.^4^ However, medical technology innovation and the development of new competitive solutions require the tight collaboration between engineers and medical doctors, which in turn is key to effective progress. This partnership is, however, hampered by scientific language barriers and historically evolved, relatively poorly matched educational curricula. At first sight, the medical education appears heavily centered on clinical experience and seniority, and authoritarian^5^ structures remain prevalent. Such configurations are described by many as central to a smooth functioning of the medical system in its entirety.^6^ Structure, guidelines, and clear responsibilities are of utmost importance, especially in time-critical situations, where fast action is crucial to provide the best possible response to clinical emergencies. Conversely, the curricula at most engineering schools focus on the development of critical thinking skills^7^ and train their graduates in challenging the established on a regular basis. Also, in academic settings, scientists and engineers are freer to prioritize and time their work, especially compared to colleagues working exclusively as physician scientists or in a clinical setting. Additionally, in contrast to their clinically working colleagues, full-time, nonclinical researchers do not have to juggle the responsibility of clinical duties and research.^8^ In the case of physician scientists, academic research is frequently done on weekends and during evening hours, once the clinical work is completed. In fact, to remain competitive, most clinicians and physician scientists use also their holidays or compensation time after night shifts to advance their research.

Thus, bringing these two communities together to work effectively, is not necessarily easy. In the following, potential strategies are presented, that might aid in facilitating better communication and enable inspiring collaborations between frontline clinicians, (physician) scientists and engineers.

## Gaining Insights by Site Visits

An easy first step is to gain insight into the daily clinical routine and education system. Some hospitals offer clinical site visits, where they introduce guests (usually representatives from pharmaceutical or medical device companies but also equally fitting for nonclinical scientists) to their different departments and the clinical workflow. During site visits, shadowing one of the lead clinicians for a couple of days is typically offered, which provides an exceptional opportunity to gain direct insight into clinical workflows and current challenges in an unfiltered setting with real-world clinical scenarios. For example, as part of a three-day visit to a breast cancer center, scientists could observe various processes and understand current obstacles encountered by clinicians and the various other stakeholders. The stay may include a visit to the mammography consultation and image-guided biopsy collection unit as well as the pathology department. Of great interest is also the participation in a tumor board meeting, where all the different domain experts (e.g., radiologists, surgeons, oncologists, pathologists) discuss and develop a therapy plan for each individual patient. Visitors may also have the opportunity to observe image-guided tumor and lymph node resections in an operating room, and experience the close collaboration between surgeons and pathologists during the surgery through real-time feedback regarding the local tumor margins.^9^ The site visit may also include an opportunity to meet patients undergoing chemotherapy, gain insights into their experience, and join the follow-up consultations with psychologists and nutritional therapists. While the visit to an oncological center might be an intense and lifelong experience for a nonclinical scientist, who we will be confronted with tough situations and terminally ill patients, it brings an entirely new perspective to their work in biomedical research. The deep understanding of the clinical reality may further inspire and motivate young engineers to develop new solutions together with the medical doctors to improve patient outcomes and increase quality of life. In addition, such visits offer unique insights into clinical practice, which, despite the exposure through social media and TV, remain challenging to grasp in its entire complexity for nonclinicians. Such site visits and hands-on experiences, including patient contact, greatly contribute toward the understanding of care pathways and environment,^4^ and the different stakeholder interests.

A site visit can also easily be reversed. Being an essential component of a collaborative approach, training opportunities in research laboratories may be adapted to a specific clinical discipline and career stage of the clinical visitor. While the involvement can vary greatly, benefits can be enormous in a wide range of scenarios, spanning from the role as a consultant, who provides critical feedback and a clinical perspective, to hands-on training and the performance of experimental research, e.g., as part of an MD–PhD program. Such low-key informal interactions on a regular basis have the potential to benefit both the clinician as well as the material scientist. Such site visits can be expanded to (compulsory) internships for engineering and medicine students and junior physicians, and have proven to be a very promising measure to lower the communication barrier. However, depending on student numbers, it may not always be feasible to integrate such internships into the curricula of engineering students with the current infrastructure and available resources. Similarly, such exchanges may also include sabbaticals of senior staff at any stage in their career. While highly beneficial, the current situation in hospitals with staff shortage, limited space and operation at maximal capacity renders such “hands-on” exchanges and internships more challenging than ever. Additional financial incentives may be put in place under the premise that patient care can be guaranteed throughout, with the hope that such exchanges positively influence the motivation and commitment of all involved staff members and eventually contribute to better, evidence-based patient care.

## Joint Ideation and Regular Feedback on Ideas and Prototypes

**Figure 1:**
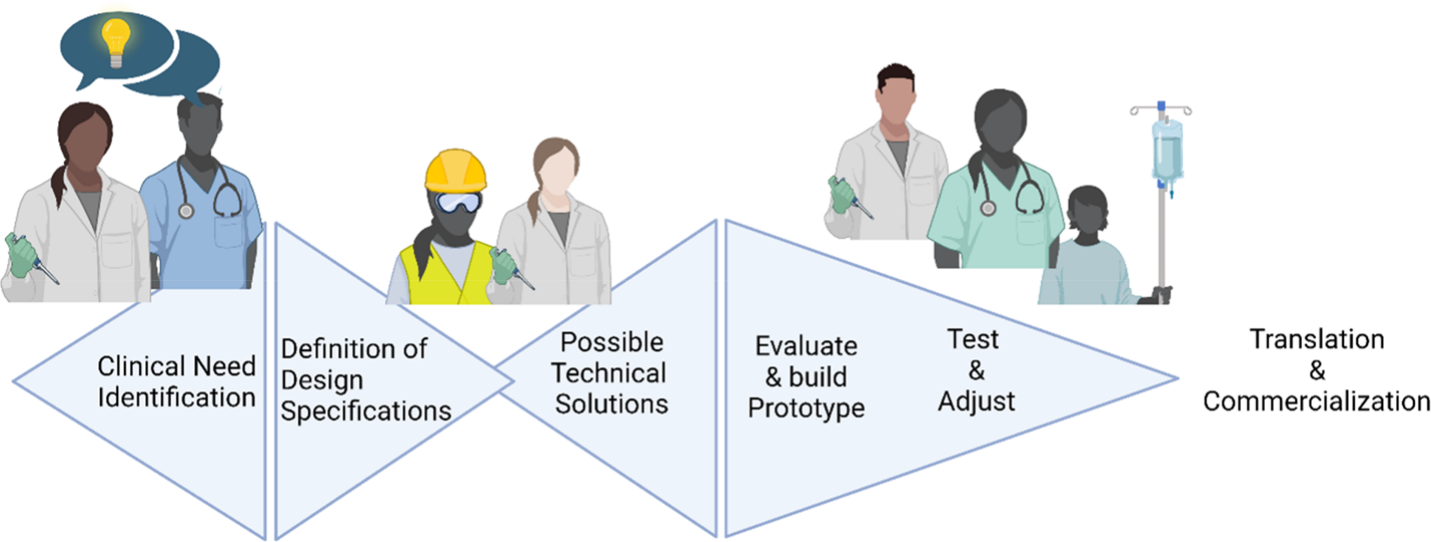
Development and implementation pathway of novel medical technology. Close collaboration between scientists, engineers, and clinicians should serve as primary strategy to accelerate disruptive medical technology innovation and protection of resources.

Spending time to listen carefully and paying attention to nuances and details is key in the identification of needs, and is not limited to the healthcare setting. To ask open questions, driven by curiosity and broad interest, greatly increases the ability to grasp the complexity of the entire situation at hand, including the various stakeholder interests. When looking for new ideas and potential research lines, attending top-quality local clinical symposia, clinical meetings and conferences, covering a wide variety of different disciplines may be a highly effective opportunity. In these interactions with clinicians (including first-year residents all the way to department chairs), questions regarding the most pressing clinical challenges they are currently facing are great way to start a conversation. Sometimes you will get to learn about shortcomings in logistics, or insurance companies not covering treatments, two aspects that are sometimes challenging to be addressed by academic researchers. Most of the time, however, the answers cover technical problems, which we as researchers and engineers may be able to help overcome. Even though, we may not necessarily have immediate competitive solutions in mind, we collect various clinical needs and document them carefully. During such interactions, we also ask the medical doctors, if they had a wish for something a scientist or engineer could develop or build for them, or together with them. “What would it be and what could be an ideal approach?” are only two key questions. To identify ideas, potential solutions and current boundaries or maybe even “no-goes” (technical, logistical, or ethical) is of great importance to establish collaborations and to work toward a common goal in a resourceful and sustainable way. After returning to the lab, obtained information is filtered and classified regarding clinical and general relevance, considering already available solutions, either commercialized or in development. The scientific literature and the patent space, typically based on an assisted patent search, are also carefully checked. If all the aforementioned looks promising and the clinical problem is deemed important, we enter the ideation phase of a design thinking approach,^10^ frequently together with an interdisciplinary team, covering medicine, biology, chemistry, engineering, computer science, physics and mathematics. We implement the development pathway and discuss potential approaches and technical solutions in the broadest and most open way possible (Figure 1). Then we analyze their pros and cons in close iteration with the clinical collaborators, and whether we have the required skills to design and develop (prototype and test) the solution available in the lab (or if we can acquire them through collaboration). We seek to get broad and repeated feedback on the research progress, present the approach at leading conferences of the medical discipline and decide together on the next steps and the most convincing proof-of-concept experiments (basic and preclinical). The constant feedback from clinical partners typically greatly improves the competitiveness of innovations and the amenability for the translation to clinics, and the acceptance by the main stakeholders. Additionally, in our experience, such close interactions have sparked a great number of new collaborations and opened new research avenues.

## Commit and Create Situations, Where All Stakeholders Win

Innovating new materials and technologies for healthcare obviously requires an enormous commitment and dedication from both, scientists/engineers and clinical partners. In our experience, it is of tremendous importance to design projects (and publications) in such a way that both parties equally benefit, considering their individual contribution. Unfortunately, the current system of publications does not facilitate such settings, because for the top-tier journals an article typically covers the entire chain, from material design and innovation to preclinical efficacy and safety. Especially in surgical sciences, the performance of high-quality, preclinical animal studies requires an enormous commitment from surgeons and pathologists to obtain and analyze the tissues properly. Designing a clinically oriented study for a high-ranking specialty journal in addition to a more material-centered study might be an attractive strategy creating rewards for both, the basic research group and the clinical partner. At the same time, such a publication approach has the benefit of achieving high visibility in both, the community of material sciences, and the leading clinical journals, read by most clinicians in the respective field. It is also important to recognize that strong publication records are of high importance for aspiring clinical leaders. Academically interested young clinicians have a fairly small time-window in their career to build such a publication track record, and this has frequently to be achieved while undergoing clinical training (including night shifts) at the same time. Recognizing these boundaries and creating opportunities for junior scientists to collaborate may greatly increase the commitment and the creativity of the entire team. Also, the establishment of funding schemes dedicated to support cross-disciplinary exchange and training have been put in place in many countries (e.g., joint calls between the EPSRC and BBSRC/MRC in the UK and cross-disciplinary calls by the NIH in the US); however, an ideal balance between imposed constraints (on the applicant’s qualification and seniority, and area of expertise) and scientific creativity and quality has yet to be found in order to identify project with maximal impact and clinical utility.

## Promote and Support Young Talents and Diversity

In addition to the creation of an open, inclusive and interdisciplinary research environment, we also strongly encourage the implementation of such collaborative approaches early into the educational engineering curricula, e.g., in the form of project-based learning. Ideally, student teams should contain mixed backgrounds, including medical students, biologists, chemists, engineers, physicists and computer scientists, and ideally also students with economics background. In our experience, project-based learning in interdisciplinary groups, to solve a real engineering challenge (Table 1) based on design thinking, is a hugely fulfilling experience for students and group tutors alike. Such projects offer students the opportunity to learn multiple aspects, which are only poorly covered by the conventional curriculum (including teamwork, creative thinking without too many boundary conditions, structure and organization, project management). Above all, it is potentially a very rewarding experience. In cases where an innovative solution is found, such projects may even be pursued further and result in a start-up. In fact, the push through the development phase and regulatory approval requires expertise and major funding, and is not typically a focus of academic research laboratories or clinical centers. An adequate institutional and governmental support system for (student-initiated) start-ups, and the increasing recognition of entrepreneurship as an attractive career opportunity, might at least partially aid in overcoming the translational gap. Additionally, start-ups may be the right tool for the advancement of new technologies to a stage where a partnership with a larger company becomes feasible. Such partnerships are relatively rare for early stage technologies in the medtech and pharma sector, and maturation of the technology is typically only achievable under the umbrella of a start-up. Once a first clinical proof-of-principle has been achieved, partnerships with industry are instrumental for scale-up and establishment on the market. Maximizing collaboration between stakeholders from academia, industry, governmental institutions and the regulatory agencies is highly beneficial for the successful technology transfer so that the benefits can ultimately be realized for patients.^11^

**Table 1:** Student Innovation: Sample Topics for Interdisciplinary Innovation Challenges

examples for potential interdisciplinary innovation challenge topics
preventing sepsis deaths
avoiding late-stage cancer diagnoses
overcoming organ shortage
preventing medical device-associated infections
extending the healthspan (the period of one’s life that someone is healthy)
enabling human augmentation through technology (“superhumans”)

## Conclusions

Close collaboration of engineers and scientists with clinical partners and physician scientists is key to sustainable innovation in medicine. Critical evaluation of the developed technical solution (as well as alternatives) at every stage in the development cycle and beyond is of utmost importance in order to ensure maximal impact and responsible and sustainable use of resources. Most importantly, it gives direct purpose to the work of engineers, and offers a continuous source for inspiration, cross-fertilization and motivation. Promotion of diversity in teams in every aspect and investment in overcoming communication barriers are instrumental to maximize creativity, innovation and impact for progressive healthcare and medical technology of the future.

## References

[ref1] Council N. R. Materials in the New Millennium: Responding to Society’s Needs; National Academies Press, 2001.

[ref2] Chakraborty M.; Jain S.; Rani V. Nanotechnology: Emerging Tool for Diagnostics and Therapeutics. Applied biochemistry and biotechnology 2011, 165 (5), 1178–1187. 10.1007/s12010-011-9336-6. 21847590

[ref3] Chen Y.-W.; Tanaka S.; Howlett R.; Jain L. Innovation in Medicine and Healthcare; Springer, 2021.

[ref4] Andrews R.; Greasley S.; Knight S.; Sireau S.; Jordan A.; Bell A.; White P. Collaboration for Clinical Innovation: A Nursing and Engineering Alliance for Better Patient Care. Journal of Research in Nursing 2020, 25 (3), 291–304. 10.1177/1744987120918263. 34394638 PMC7932479

[ref5] Vanstone M.; Grierson L. Thinking about Social Power and Hierarchy in Medical Education. Medical Education 2022, 56 (1), 91–97. 10.1111/medu.14659. 34491582

[ref6] Salehi P. P.; Jacobs D.; Suhail-Sindhu T.; Judson B. L.; Azizzadeh B.; Lee Y. H. Consequences of Medical Hierarchy on Medical Students, Residents, and Medical Education in Otolaryngology. Otolaryngol. Head Neck Surg. 2020, 163 (5), 906–914. 10.1177/0194599820926105. 32482121

[ref7] Claris L.; Riley D. Situation Critical: Critical Theory and Critical Thinking in Engineering Education. Engineering Studies 2012, 4 (2), 101–120. 10.1080/19378629.2011.649920.

[ref8] Morel P. A.; Ross G. The Physician Scientist: Balancing Clinical and Research Duties. Nature Immunology 2014, 15 (12), 1092–1094. 10.1038/ni.3010. 25396341

[ref9] Butler-Henderson K.; Lee A. H.; Price R. I.; Waring K. Intraoperative Assessment of Margins in Breast Conserving Therapy: A Systematic Review. Breast 2014, 23 (2), 112–119. 10.1016/j.breast.2014.01.002. 24468464

[ref10] Oliveira M.; Zancul E.; Fleury A. L. Design Thinking as an Approach for Innovation in Healthcare: Systematic Review and Research Avenues. BMJ. Innovations 2021, 7 (2), 491–498. 10.1136/bmjinnov-2020-000428.

[ref11] Rösslein M.; Liptrott N. J.; Owen A.; Boisseau P.; Wick P.; Herrmann I. K. Sound Understanding of Environmental, Health and Safety, Clinical, and Market Aspects Is Imperative to Clinical Translation of Nanomedicines. Nanotoxicology 2017, 11 (2), 147–149. 10.1080/17435390.2017.1279361. 28055261

